# Multi-Omics Analysis of Acute Lymphoblastic Leukemia Identified the Methylation and Expression Differences Between BCP-ALL and T-ALL

**DOI:** 10.3389/fcell.2020.622393

**Published:** 2021-01-21

**Authors:** Jin-Fan Li, Xiao-Jing Ma, Lin-Lin Ying, Ying-hui Tong, Xue-ping Xiang

**Affiliations:** ^1^Department of Pathology, The Second Affiliated Hospital, School of Medicine, Zhejiang University, Hangzhou, China; ^2^Department of Pharmacy, Cancer Hospital of the University of Chinese Academy of Sciences (Zhejiang Cancer Hospital), Institute of Cancer and Basic Medicine (IBMC), Chinese Academy of Sciences, Hangzhou, China

**Keywords:** acute lymphoblastic leukemia, Boruta, Monte Carlo feature selection, network analysis, hub, multi-omics, expression, methylation

## Abstract

Acute lymphoblastic leukemia (ALL) as a common cancer is a heterogeneous disease which is mainly divided into BCP-ALL and T-ALL, accounting for 80–85% and 15–20%, respectively. There are many differences between BCP-ALL and T-ALL, including prognosis, treatment, drug screening, gene research and so on. In this study, starting with methylation and gene expression data, we analyzed the molecular differences between BCP-ALL and T-ALL and identified the multi-omics signatures using Boruta and Monte Carlo feature selection methods. There were 7 expression signature genes (CD3D, VPREB3, HLA-DRA, PAX5, BLNK, GALNT6, SLC4A8) and 168 methylation sites corresponding to 175 methylation signature genes. The overall accuracy, accuracy of BCP-ALL, accuracy of T-ALL of the RIPPER (Repeated Incremental Pruning to Produce Error Reduction) classifier using these signatures evaluated with 10-fold cross validation repeated 3 times were 0.973, 0.990, and 0.933, respectively. Two overlapped genes between 175 methylation signature genes and 7 expression signature genes were CD3D and VPREB3. The network analysis of the methylation and expression signature genes suggested that their common gene, CD3D, was not only different on both methylation and expression levels, but also played a key regulatory role as hub on the network. Our results provided insights of understanding the underlying molecular mechanisms of ALL and facilitated more precision diagnosis and treatment of ALL.

## Introduction

Acute lymphoblastic leukemia (ALL) as a common cancer is a heterogeneous disease that originates from lymphocyte progenitor cells of B-cells or T-cells. It is a childhood malignant tumor that comprises >25% of pediatric neoplasia in American (Jabbour et al., 2015; Pui et al., 2015). Among adults, the incidence of ALL is much lower, accounting for only 0.2% of all cancers. However, the prognosis of ALL remains worrying, with an estimated 5-year overall survival (OS) of between 20 and 40% (Sive et al., 2012; Wolach et al., 2017). According to the World Health Organization (WHO) classification, ALL can be divided into B-cell ALL (B-ALL) and T-cell ALL (T-ALL). B-cell precursor ALL (BCP-ALL) is one of the B-ALL (Herold et al., 2014; Jones et al., 2016). In children’s ALL, it is mainly divided into BCP-ALL and T-ALL, accounting for 80–85% and 15–20%, respectively (Graux, 2011). These different subtypes are characterized by structural chromosomal rearrangements and repeated copy number alterations, which with great clinical significance (Goldberg et al., 2003).

There are prognosis, treatment and genetics differences between BCP-ALL and T-ALL (Gutierrez et al., 2014; Pui et al., 2015): (1) The prognosis of T-ALL patients is always worse than BCP-ALL patients (Goldberg et al., 2003; Eckert et al., 2013); (2) Many targeted immunotherapies have been developed for BCP-ALL patients but not for T-ALL patients (Pui et al., 2015); (3) T-ALL is associated with a wide range of acquired genetic abnormalities, which leads to abnormal proliferation and development stagnation of malignant lymphoid progenitor cells (Van Vlierberghe et al., 2008; Teitell and Pandolfi, 2009). This poses a challenge to the development of targeted therapy with wide application value. In the studies of the gene expression profile of ALL, the high expression of CD45 in leukemia cells was not only related to the poor prognosis of BCP-ALL patients but also to the poor prognosis of T-ALL patients. However, the prognostic correlation of CD45 expression in T-ALL was much higher than that in BCP-ALL (Hermiston et al., 2003; Cario et al., 2014). Moreover, PR-104 has been shown to specifically target hypoxic regions of leukemia infiltration, and was effective in the treatment of T-ALL xenotransplantation, but not in the treatment of BCP-ALL xenograft (Benito et al., 2011).

In this study, starting with methylation and gene expression data, we analyzed the molecular differences between BCP-ALL and T-ALL, screened out the molecular characteristics, and explored the relationship between these characteristics and the two subtypes of ALL.

## Materials and Methods

### The Multi-Omics Dataset of ALL

We downloaded the methylation and expression data of 69 BCP-ALL and 30 T-ALL patients from GEO (Gene Expression Omnibus) under accession number of GSE49031 and GSE47051 (Nordlund et al., 2013, 2015; Borssen et al., 2018), respectively. It was a large study performed by Uppsala University. There were originally 945 methylation samples and 108 expression samples. But the overlapped sample size between methylation data and expression data was 99 and within the 99 samples, there were 69 BCP-ALL and 30 T-ALL patients. Our goal was to systematically investigate the molecular differences between BCP-ALL and T-ALL and try to use these molecular differences to explain the clinical differences.

The methylation data were generated with Illumina HumanMethylation450 BeadChip and there were 485,577 methylation probes. Since there were missing values, we filtered the probes with missing values in at least 20% samples and kept 485,096 probes. Since the probes out of gene ranges were hard to explain, we kept the 317,845 probes that can be annotated onto genes and imputed the missing values using KNN (K = 10) method. Meanwhile, the expression data were generated with Affymetrix Human Genome U133 Plus 2.0 Array. The expression values of probes corresponding to the same gene were averaged. At last, the dataset was the expression levels of 15,888 genes and methylation levels of 317,845 probes in 69 BCP-ALL and 30 T-ALL patients.

### Filter the Irrelevant Features Using Boruta

As we mentioned before, there were 15,888+317,845 = 333,733 features for each ALL sample. The number of features was much larger than the sample size. If we directly analyze all these 333,733 features, there will be too much noise and too many random feature combinations that can classify the samples. Therefore, we filtered the irrelevant features using Boruta method (Kursa and Rudnicki, 2010). The Boruta method can find out the relevant features and significantly reduce the number of features based on ensemble learning of random forest classifiers. Boruta is a widely used method and has been proven to be an effective method to find all relevant features (Pan et al., 2020; Yuan et al., 2020; Zhang et al., 2020).

### Identify the Important Features Using Monte Carlo Feature Selection

Although Boruta method can filter irrelevant features and keep the relevant features, usually the number of features was still too large and the importance of features were still unknown. We need more sophisticated feature selection method to calculate the importance of features and rank the features. In this study, we applied MCFS (Monte Carlo Feature Selection) (Draminski et al., 2008). The MCFS has been widely used for feature selection (Chen et al., 2018, 2019; Pan et al., 2018, 2019a,b; Li et al., 2020). It divided the whole dataset into many small subsets. The subsets had much less features and the data structure of these subsets were relatively simple. Decision trees can be easily constructed. Based on all the trees on all the subsets, the importance of each feature can be calculated. The basic idea was that if a feature appeared in many trees, it was important and if a feature can classify many samples correctly, it was important. Based on these two rules, the importance of each feature was calculated. What’s more, the data was shuffled to generate random importance of each feature, the significance of each feature can be estimated by comparing the random importance and actual importance. At last, the significant features with importance much greater than permutated importance can be selected. Meanwhile, the RIPPER (Repeated Incremental Pruning to Produce Error Reduction) rules within the trees can be cross-validated and their accuracy can be estimated.

## Results and Discussion

### The Relevant Features Identified by Boruta

As we mentioned there were 333,733 features (15,888 expression feature and 317,845 methylation features) for each ALL sample. The number of features were much larger than the sample size (99 in this study). Most of the features were not relevant to ALL. Keeping such features in the dataset will introduce noise and make the analysis inaccurate. Therefore, we adopted Boruta method (Kursa and Rudnicki, 2010) to remove irrelevant features. After running Boruta, 1,398 features were kept. Within these 1,398 features, there were 1,374 methylation features and 24 expression features.

### The Important Features Identified by MCFS

The number of features filtered by Boruta (1,398) was still too large to be biomarkers. Therefore, we further reduced the number of features with MCFS method and finally identified 175 significant features. Within the 175 features, there were 168 methylation features (probe IDs starting with “cg”) and 7 expression features (CD3D, VPREB3, HLA-DRA, PAX5, BLNK, GALNT6, SLC4A8). These 175 features were given in Table 1. The annotations of the 168 methylation probes of in Supplementary Table 1.

**TABLE 1:** The 175 important features identified by MCFS.

Rank	Feature	Rank	Feature	Rank	Feature	Rank	Feature	Rank	Feature
1	cg26547698	36	cg19365697	71	cg00262446	106	cg06786219	141	cg20278269
2	CD3D	37	cg11086982	72	cg11071448	107	cg23387468	142	cg27627006
3	cg04690998	38	cg23275914	73	cg15188623	108	cg27280688	143	cg09203501
4	VPREB3	39	cg01391022	74	cg07255197	109	cg02368508	144	cg24690709
5	cg26437842	40	cg01686739	75	cg25468516	110	cg04715649	145	cg02673417
6	cg09740560	41	cg10746778	76	cg01582937	111	cg19610383	146	cg27263049
7	cg18085400	42	cg06560887	77	cg11139102	112	cg22056218	147	cg18245281
8	cg02891579	43	cg06876053	78	cg04473078	113	cg01290568	148	cg01467417
9	cg24710886	44	cg22051146	79	cg17355865	114	cg10253457	149	cg12971694
10	cg05998426	45	cg06571407	80	cg13948857	115	cg26833538	150	cg13804478
11	HLA-DRA	46	cg19785066	81	cg02655351	116	cg09976369	151	cg17984638
12	cg09773499	47	cg04346861	82	cg08874645	117	cg26795340	152	cg19844326
13	cg24999105	48	cg23379806	83	cg19843939	118	cg11963912	153	cg24864097
14	cg26607748	49	cg00004667	84	cg03364781	119	cg20464143	154	cg22628286
15	cg09983897	50	cg02334109	85	cg05524458	120	cg02297801	155	cg11321459
16	cg25620356	51	cg27021986	86	cg24937136	121	cg07003587	156	cg14989202
17	cg01731685	52	cg00231528	87	cg02574101	122	cg22905350	157	cg13094252
18	cg00661777	53	cg08894788	88	cg19006008	123	cg05115424	158	cg11348106
19	cg13031167	54	cg09897604	89	cg22232207	124	cg13482010	159	cg12960305
20	cg08146609	55	cg09864245	90	cg13767306	125	cg15662251	160	cg19339902
21	cg26121730	56	cg10156042	91	cg14788673	126	cg15897310	161	cg06560379
22	cg22881247	57	cg26574610	92	PAX5	127	cg14251777	162	cg00739471
23	cg14913610	58	cg22964469	93	cg02022181	128	cg03145274	163	SLC4A8
24	cg00120948	59	cg20934596	94	cg20117103	129	cg07151443	164	cg26262049
25	cg09285418	60	cg05533539	95	cg19750657	130	GALNT6	165	cg05276137
26	cg01937819	61	cg10142436	96	cg07545925	131	cg01278291	166	cg17398227
27	cg20907136	62	cg19140262	97	cg12577411	132	cg08995609	167	cg16324306
28	cg14499058	63	cg10789956	98	cg20090290	133	cg22996440	168	cg03437770
29	cg08347042	64	cg14590369	99	cg23616139	134	cg19921353	169	cg08854008
30	cg04926556	65	cg00310940	100	cg08187585	135	cg01591579	170	cg14154784
31	cg07217499	66	cg01595717	101	BLNK	136	cg09578155	171	cg01456517
32	cg18696027	67	cg27531366	102	cg16824282	137	cg12763828	172	cg02709032
33	cg03100639	68	cg07786657	103	cg02625929	138	cg01176028	173	cg03802696
34	cg09989037	69	cg04370174	104	cg10591771	139	cg27036638	174	cg06164961
35	cg06132620	70	cg10131232	105	cg02056653	140	cg26396492	175	cg12024826

As we mentioned in section “Methods,” the MCFS method can also extract the classification rules. The confusion matrix of these RIPPER classification rules evaluated with 10-fold cross validation repeated 3 times was given in Table 2. The overall accuracy, accuracy of BCP-ALL, accuracy of T-ALL were 0.973, 0.990, and 0.933, respectively. These results meant that these features can classify the BCP-ALL and T-ALL very well.

**TABLE 2:** The confusion matrix of the RIPPER rules evaluated with 10-fold cross validation repeated 3 times.

	Predicted BCP-ALL	Predicted T-ALL
Actual BCP-ALL	205	2
Actual T-ALL	6	84

### The Enrichment Analysis of the Selected Genes

**FIGURE 1:**
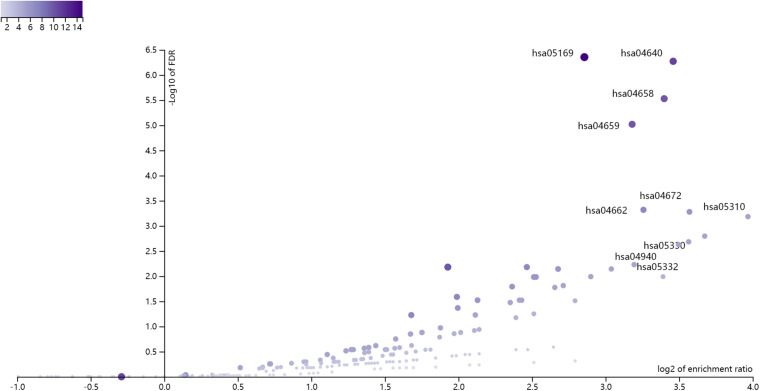
The enrichment results of the 180 selected genes using WebGestalt. The x axis was log2 of enrichment ratio while the y axis was the -Log10 of FDR. The pathways on the top right corner were the significantly enrich pathways. It can be seen that hsa04640 Hematopoietic cell lineage was the enriched KEGG pathway.

Based on the annotations in Supplementary Table 1, we mapped the 168 methylation probes onto 175 genes. There were two overlapped genes (CD3D and VPREB3) between the 175 methylation signature genes and the 7 expression signature genes. We combined the 175 methylation signature genes and the 7 expression signature genes. Since there were two overlapped genes between them, there were 180 selected genes. We enriched the 180 selected genes onto KEGG pathways using WebGestalt^1^ (Wang et al., 2017). The KEGG enrichment results were shown in Figure 1. The x axis was log2 of enrichment ratio while the y axis was the -Log10 of FDR. The pathways on the top right corner were the significantly enrich pathways. It can be seen that hsa04640 Hematopoietic cell lineage was the enriched KEGG pathway. The were 11 selected genes on hsa04640 Hematopoietic cell lineage pathway: CD3D, CD3E, CD3G, CD59, FCER2, GP9, HLA-DMA, HLA-DPA1, HLA-DPB1, HLA-DRA and IL1B. The enrichment p value and FDR were 3.28e-9 and 5.35e-7, respectively. Its enrichment ratio was 11. As CD3D was dysfunctional on both methylation and gene expression levels, HLA-DRA was dysfunctional on gene expression levels and other genes were dysfunctional on methylation levels, the hsa04640 Hematopoietic cell lineage pathway was dysfunctional on both methylation and gene expression levels.

### The Network of Methylation and Expression Signature Genes

**FIGURE 2:**
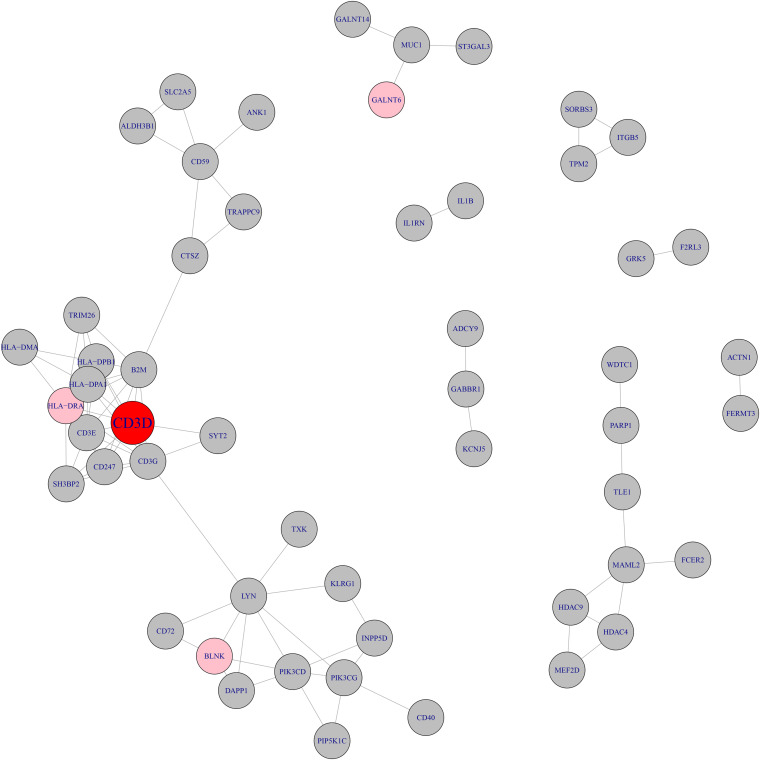
The network of methylation and expression signature genes. The methylation and expression signature genes were colored in gray and pink respectively. The red node, CD3D, was both methylation signature gene and expression signature gene. CD3D was the hub node of the network.

We searched the methylation and expression signature genes in STRING database^2^ (Szklarczyk et al., 2019) and their network with highest confidence (confidence score >0.900) was shown in Figure 2. The confidence score integrated the information from multiple sources including text mining, experiments, databases, co-expression, neighborhood, gene fusion and co-occurrence. It ranged from 0 to 1. The higher the confidence score was, the more reliable the interaction was. The cutoff of confidence score was set to be 0.900 since 0.900 was considered to be highest confidence in the STRING database. It can be seen that CD3D was the hub of the whole network. CD3D and another neighbor gene on the network, HLA-DRA, both belonged to hsa04640 Hematopoietic cell lineage pathway. The protein encoded by CD3D is part of the T cell receptor / CD3 complex (TCR/CD3 complex) and is involved in T cell development and signal transduction (Shi et al., 2019). CD3D has been shown to work with PKRCQ as a model to distinguish between B-ALL and T-ALL (Ma et al., 2016).

### The Functional Analysis of the Selected Genes

Within the 7 expression signature genes, beside CD3D which was discussed above, VPREB3 and HLA-DRA also looked promising.

**VPREB3** is the B-cell receptor component and its overexpression can activate the pro-survival PI3K pathway (Soldini et al., 2014). It has been reported as a biomarker for B-cell lymphoma by many studies (Heerema-McKenney et al., 2010; Rodig et al., 2010; Soldini et al., 2014).

**HLA-DRA** is related to the antigen presentation steps of the immune system (Hotchkiss et al., 2013). In the study of Morrison et al. (2010), women and children with multiple sclerosis (MS) had a fourfold increased risk of developing ALL. And, there was a certain correlation between MS and HLA-DRA single nucleotide polymorphism (SNP) (Morrison et al., 2010). Moreover, HLA genes are candidate genetic susceptibility loci for childhood ALL, HLA-DP1 was significantly correlated with ALL in children (Urayama et al., 2012). According to Ross et al. (2019), the ablation of POZ domain of ZBTB17 (Miz-1) interferes with its interaction with c-MYC and delays the occurrence of T-ALL and B-ALL.

Within the 175 methylation signature genes, there were many great candidates, such as HDAC4, HDAC9, LMO2, MEF2D, CD40, PAX5, BLNK and TLE1.

**HDAC4** and **HDAC9** are Histone deacetylases (HDACs) which may be a potential target for cancer treatment, including hematological malignancies. Moreno et al. (2010) detected the expression profile of HDAC gene in ALL samples by PCR. It was found that HDAC1 and HDAC4 were highly expressed in T-ALL and HDAC5 was highly expressed in B-ALL. Moreover, the expression of HDAC9 was correlated with B-ALL patients (Moreno et al., 2010).

**LMO2** plays an essential role during early hematopoiesis and is frequently activated in T-ALL patients (Morishima et al., 2019). Wu et al. have deeply studied the mechanism of LMO2 in T-ALL and found that LMO2 can induce the transcriptional inhibition of ZEB1, while ZEB1 plays an important role in promoting T cell differentiation and may play an anti-cancer role in T-ALL (Wu et al., 2018). Several studies have also confirmed that LMO2 plays an important role in T-ALL (Curtis and McCormack, 2010; Homminga et al., 2012; Rahman et al., 2017).

**MEF2D** has been reported as a biomarker for a B-ALL subtype with a low survival rate. According to Zhang M et al., MEF2D-SS18 fusion gene blocks the differentiation of B cells, which plays an important role in the pathogenesis and prognosis of B-ALL (Zhang et al., 2018). Besides, Suzuki et al. (2016) confirmed that MEF2D-BCL9 fusion gene is associated with juvenile acute BCP-ALL.

**CD40** is the member of the tumor necrosis factor receptor (TNFR) family, are critical regulators of lymphocyte growth and differentiation. Troeger et al. (2008) confirmed that the high expression of CD40 in BCP-ALL cells is an independent prognostic indicator, which indicates a better recurrence-free survival.

**PAX5** is a haplotype tumor suppressor gene in human B-All, which is involved in a variety of chromosome translocation (Jamrog et al., 2018). In the investigation and analysis of Bastian et al. (2019), it was found that the army of patients with BCP-ALL subgroup carried PAX5 mutation.

**BLNK** is an adapter molecule essential to the development of normal B cells and is associated with increased pro-B/pre-B-cell expansion in mice. It was reported that BLNK deficiency was one of the main causes of B-ALL (Imai et al., 2004). The results of Nakayama et al. suggested that somatic loss of BLNK and concomitant mutations leading to constitutive activation of Jak/STAT5 pathway result in the generation of BCP-ALL (Nakayama et al., 2009).

**TLE1** can be used as an indicator of poor prognosis of T-ALL (Brassesco et al., 2018) and the expression of ATP10A was up-regulated in BCP-ALL (Olsson et al., 2014).

## Conclusion

Although there have been studies on the clinical differences between BCP-ALL and T-ALL, there has been no in-depth study of their underlying mechanism. In our study, the multi-omics profiles in BCP-ALL and T-ALL were analyzed. The discovered epigenetic changes of ALL and their possible effects on gene expression can help us understand the molecular mechanisms of the development, progression and recurrence of ALL. In ALL, those molecular characteristics have the function of differential diagnosis, targeted therapy and so on. At the same time, our research not only provides new information about the methylation and gene expression pattern of ALL, but also provides a selective reference for the study of ALL genes and methylation sites.

## Data Availability Statement

The original contributions presented in the study are included in the article/Supplementary Material, further inquiries can be directed to the corresponding author/s.

## Author Contributions

J-FL, X-pX, and X-JM contributed to the study design. L-LY conducted the literature search. Y-hT, J-FL, and X-JM acquired the data. J-FL and X-pX wrote the article. X-JM performed data analysis. J-FL and L-LY revised the article and gave the final approval of the version to be submitted. All authors read and approved the final manuscript.

## Conflict of Interest

The authors declare that the research was conducted in the absence of any commercial or financial relationships that could be construed as a potential conflict of interest.

## Footnotes

1 http://www.webgestalt.org

2 http://string-db.org/

## Supplementary Material

Supplementary Table 1 The annotations of the 168 methylation features.
